# Crystal structure of (2*R*)-1-[(methyl­sulfon­yl)­oxy]propan-2-aminium chloride: a chiral mol­ecular salt

**DOI:** 10.1107/S2056989015015972

**Published:** 2015-09-12

**Authors:** H. R. Rajegowda, B. S. Palakshamurthy, N. K. Lokanath, S. Naveen, P. Raghavendra Kumar

**Affiliations:** a Department of Studies and Research in Chemistry, Tumkur University, Tumkur 572 103, Karnataka, India; b Department of Studies and Research in Physics, U.C.S., Tumkur University, Tumkur, Karnataka 572 103, India; c Department of Studies in Physics, University of Mysore, Manasagangotri, Mysore, Karnataka 570 005, India; d Institution of Excellence, University of Mysore, Manasagangotri, Mysore 570 006, India

**Keywords:** crystal structure, chiral methane­sulfonate, hydrogen bonding, salt

## Abstract

In the title chiral mol­ecular salt, C_4_H_12_NO_3_S^+^·Cl^−^, the cation is protonated at the N atom, producing [*R*NH_3_]^+^, where *R* is CH_3_SO_2_OCH_2_C(H)CH_3_. The N atom in the cation is *sp* ^3^-hybridized. In the crystal, cations and anions are connected by strong N—H⋯Cl hydrogen bonds to generate edge-shared 12-membered rings of the form {⋯Cl⋯HNH}_3_. This pattern of hydrogen bonding gives rise to zigzag supra­molecular layers in the *ab* plane. The layers are connected into a three-dimensional architecture by C—H⋯O hydrogen bonds. The structure was refined as an inversion twin.

## Related literature   

For background to chiral 2-amino-2-(alk­yl/ar­yl/aralk­yl)ethyl methane­sulfonate hydro­chlorides, see: Braghiroli & Di Bella (1996 ▸); Higashiura *et al.* (1989 ▸); Morgan *et al.* (1991 ▸); Pollack *et al.* (1989 ▸); Xu (2002 ▸).
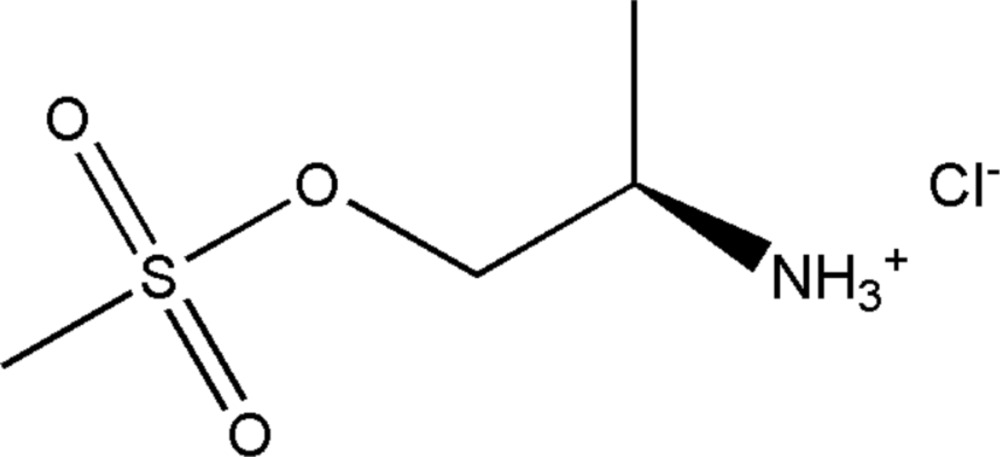


## Experimental   

### Crystal data  

C_4_H_12_ClNO_3_S^+^·Cl^−^
*M* *_r_* = 189.66
Monoclinic, 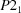

*a* = 5.4012 (1) Å
*b* = 8.2178 (2) Å
*c* = 10.2713 (2) Å
β = 94.534 (1)°
*V* = 454.48 (2) Å^3^
*Z* = 2
Cu *K*α radiation
μ = 5.57 mm^−1^
*T* = 296 K
0.24 × 0.20 × 0.16 mm

### Data collection  

Bruker APEXII CCD diffractometer
Absorption correction: multi-scan (*SADABS*; Bruker, 2013 ▸) *T*_min_ = 0.302, *T*_max_ = 0.410
2476 measured reflections
1387 independent reflections
1385 reflections with *I* > 2σ(*I*)
*R*_int_ = 0.029

### Refinement  

*R*[*F* ^2^ > 2σ(*F* ^2^)] = 0.031
*wR*(*F* ^2^) = 0.078
*S* = 1.11
1387 reflections
96 parameters
1 restraint
H-atom parameters constrained
Δρ_max_ = 0.29 e Å^−3^
Δρ_min_ = −0.42 e Å^−3^
Absolute structure: Refined as an inversion twin
Absolute structure parameter: 0.08 (3)

### 

Data collection: *APEX2* (Bruker, 2013 ▸); cell refinement: *SAINT* (Bruker, 2013 ▸); data reduction: *SAINT*; program(s) used to solve structure: *SHELXS97* (Sheldrick, 2008 ▸); program(s) used to refine structure: *SHELXL2014* (Sheldrick, 2015 ▸); molecular graphics: *Mercury* (Macrae *et al.*, 2008 ▸); software used to prepare material for publication: *SHELXL2014*.

## Supplementary Material

Crystal structure: contains datablock(s) I. DOI: 10.1107/S2056989015015972/tk5365sup1.cif

Structure factors: contains datablock(s) I. DOI: 10.1107/S2056989015015972/tk5365Isup2.hkl

Supporting information file. DOI: 10.1107/S2056989015015972/tk5365Isup3.cml

**Fig. 1.**
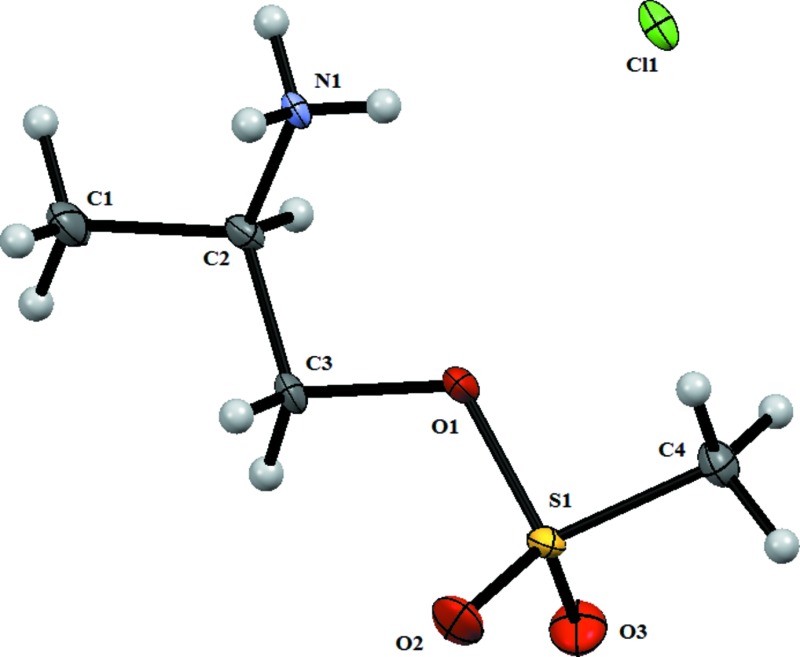
Molecular structure of the title molecular salt showing displacement ellipsoids drawn at the 50% probability level.

**Fig. 2.**
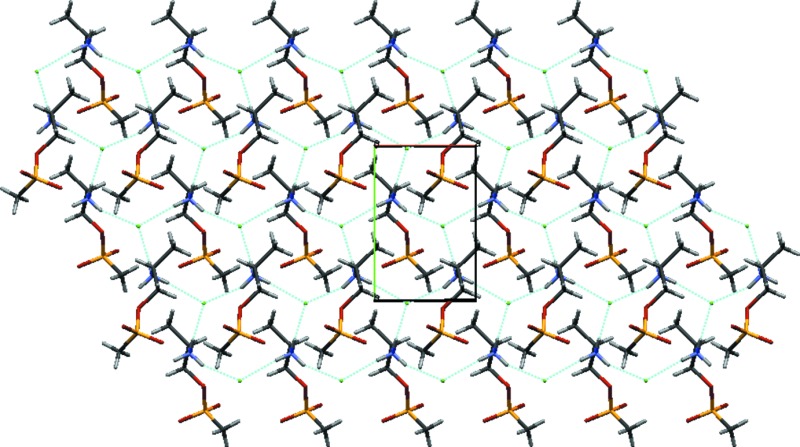
The molecular packing of the title molecular salt with N—H···Cl hydrogen bonds (aqua bonds) leading to a supramolecular assembly in the ab plane.

. DOI: 10.1107/S2056989015015972/tk5365fig1.tif
Mol­ecular structure of the title mol­ecular salt showing displacement ellipsoids drawn at the 50% probability level.

ab . DOI: 10.1107/S2056989015015972/tk5365fig2.tif
The mol­ecular packing of the title mol­ecular salt with N—H⋯Cl hydrogen bonds (aqua bonds) leading to a supra­molecular assembly in the *ab* plane.

CCDC reference: 1420721

Additional supporting information:  crystallographic information; 3D view; checkCIF report

## Figures and Tables

**Table 1:** Hydrogen-bond geometry (, )

*D*H*A*	*D*H	H*A*	*D* *A*	*D*H*A*
N1H1*E*Cl1	0.89	2.33	3.169(3)	156
C3H3*A*O3^i^	0.97	2.58	3.428(4)	147
N1H1*D*Cl1^ii^	0.89	2.24	3.116(3)	169
N1H1*F*Cl1^iii^	0.89	2.26	3.139(3)	171
C2H2*A*O2^iv^	0.98	2.44	3.186(4)	133
C4H4*B*O3^v^	0.96	2.50	3.250(4)	135
C4H4*C*O2^vi^	0.96	2.51	3.438(5)	163

Symmetry codes: (i) 
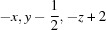
; (ii) 
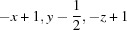
; (iii) 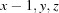; (iv) 
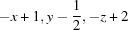
; (v) 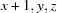; (vi) 
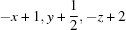
.

## supplementary crystallographic information

### S1. Chemical context

The chiral 2-amino-2-(alkyl/aryl/aralkyl)­ethyl methane­sulfonate hydro­chlorides are useful starting materials for the preparation of amines, benzoates, thio­benzoates, sulfonic acids, etc., as methane­sulfonate is a very good leaving group in nucleophilic substitution reactions. The chiral 2-(alkyl/aryl/aralkyl)­ethane­sulfonic acid derivatives and sulfono­peptides (Higashiura *et al.*, 1989) occur in high concentrations in many mammalian tissues. These compounds are involved in various important physiological processes and are used as enzyme inhibitors and heptans in the development of catalytic anti-bodies (Braghiroli & Di Bella, 1996). The enanti­omers of chiral 2-(alkyl/aryl/aralkyl)­ethane­sulfonic acid derivatives mimic the hypotensive effect of taurine (2-amino­ethane­sulfonic acid), one of the most abundant amino acids in mammals that seems to exhibit a special affinity for excitable tissues, such as brain, nerve and muscle (Xu *et al.*, 2002; Pollack *et al.*, 1989; Morgan *et al.*, 1991). In particular, the title compound was used in the synthesis of chiral amines by our group and as a part of our on-going research the structure of the title compound was determined.

### S2. Structural commentary

In the title chiral molecular salt, C_4_H_12_NO_3_S^+^.Cl^-^, the N atom is protonated resulting the cation [RNH_3_]^+^ where R is CH_3_SO_2_OCH_2_CH(CH_3_)- and the anion is chloride ion [Cl]^-^. The N atom in the cation is sp^3^ hybridized and the bond angles represents that the cation has tetra­hedral structure around N (Fig. 1). In the crystal packing N—H···Cl hydrogen bonds connect ions into a supra­molecular assembly in the *ab* plane (Fig. 2 and Table 1). Further, there exist C—H···O hydrogen bonds that connect the layers into a three-dimensional architecture.

### S3. Synthesis and crystallization

The title chiral molecular salt was synthesised as per the literature procedure (Higashiura *et al.*, 1989). An aqueous solution of HCl (4 M, 12 ml) was added to a stirred solution of (2*R*)-2-[(*tert*-but­oxy­carbonyl)­amino] propyl methane­sulfonate (2.53 g, 10 mmol ) in dioxane (15 ml). The resulting mixture was stirred for a further 1 h. The solution was then concentrated under reduced pressure and the residue obtained was recrystallized from hot ethanol to afford colourless single crystals suitable for single crystal X-ray diffraction.

### S4. Refinement details

The H atom of the NH_3_ group was located in a difference map but refined with N—H = 0.89, and with *U_iso_*(H) = 1.2*U_eq_*(N). Similarly, the other H atoms were positioned with idealized geometry using a riding model with C—H = 0.96–0.98 Å, and with *U_iso_*(H) = 1.2–1.5*U_eq_*(C). The structure was refined as an inversion twin with a Flack parameter of 0.08 (3)

### Crystal data

**Table:**

C_4_H_12_ClNO_3_S^+^·Cl^−^	prism
*M**_r_* = 189.66	*D*_x_ = 1.386 Mg m^−^^3^
Monoclinic, *P*2_1_	Melting point: 354 K
Hall symbol: P 2yb	Cu *K*α radiation, λ = 1.54178 Å
*a* = 5.4012 (1) Å	Cell parameters from 830 reflections
*b* = 8.2178 (2) Å	θ = 4.3–64.7°
*c* = 10.2713 (2) Å	µ = 5.57 mm^−^^1^
β = 94.534 (1)°	*T* = 296 K
*V* = 454.48 (2) Å^3^	Prism, colourless
*Z* = 2	0.24 × 0.20 × 0.16 mm
*F*(000) = 200	

### Data collection

**Table:**

Bruker APEXII CCD diffractometer	1387 independent reflections
Radiation source: fine-focus sealed tube	1385 reflections with *I* > 2σ(*I*)
Graphite monochromator	*R*_int_ = 0.029
Detector resolution: 2.01 pixels mm^-1^	θ_max_ = 64.7°, θ_min_ = 4.3°
φ and ω scans	*h* = −6→2
Absorption correction: multi-scan (*SADABS*; Bruker, 2013)	*k* = −9→9
*T*_min_ = 0.302, *T*_max_ = 0.410	*l* = −11→12
2476 measured reflections	

### Refinement

**Table:**

Refinement on *F*^2^	Hydrogen site location: inferred from neighbouring sites
Least-squares matrix: full	H-atom parameters constrained
*R*[*F*^2^ > 2σ(*F*^2^)] = 0.031	*w* = 1/[σ^2^(*F*_o_^2^) + (0.0448*P*)^2^ + 0.0553*P*] where *P* = (*F*_o_^2^ + 2*F*_c_^2^)/3
*wR*(*F*^2^) = 0.078	(Δ/σ)_max_ < 0.001
*S* = 1.11	Δρ_max_ = 0.29 e Å^−^^3^
1387 reflections	Δρ_min_ = −0.42 e Å^−^^3^
96 parameters	Extinction correction: *SHELXL2014* (Sheldrick, 2014), Fc^*^=kFc[1+0.001xFc^2^λ^3^/sin(2θ)]^-1/4^
1 restraint	Extinction coefficient: 0.120 (8)
0 constraints	Absolute structure: Refined as an inversion twin
Primary atom site location: structure-invariant direct methods	Absolute structure parameter: 0.08 (3)
Secondary atom site location: difference Fourier map	

### Special details

**Table:**

Geometry. All e.s.d.'s (except the e.s.d. in the dihedral angle between two l.s. planes) are estimated using the full covariance matrix. The cell e.s.d.'s are taken into account individually in the estimation of e.s.d.'s in distances, angles and torsion angles; correlations between e.s.d.'s in cell parameters are only used when they are defined by crystal symmetry. An approximate (isotropic) treatment of cell e.s.d.'s is used for estimating e.s.d.'s involving l.s. planes.
Refinement. Refined as a 2-component inversion twin.

### Fractional atomic coordinates and isotropic or equivalent isotropic displacement parameters (Å^2^)

**Table:**

	*x*	*y*	*z*	*U*_iso_*/*U*_eq_	
C1	−0.0227 (7)	0.1587 (5)	0.7527 (4)	0.0204 (8)	
H1A	−0.1807	0.2029	0.7211	0.031*	
H1B	−0.0282	0.1268	0.8423	0.031*	
H1C	0.0142	0.0655	0.7013	0.031*	
Cl1	0.67754 (13)	0.51693 (10)	0.53323 (7)	0.0149 (3)	
S1	0.33444 (13)	0.70873 (9)	0.88988 (7)	0.0118 (3)	
O1	0.3212 (4)	0.5513 (3)	0.8028 (2)	0.0190 (6)	
N1	0.1849 (5)	0.3392 (4)	0.6037 (3)	0.0111 (6)	
H1D	0.2153	0.2535	0.5544	0.013*	
H1E	0.3047	0.4127	0.5982	0.013*	
H1F	0.0395	0.3828	0.5759	0.013*	
C2	0.1771 (6)	0.2864 (4)	0.7423 (3)	0.0119 (7)	
H2A	0.3383	0.2394	0.7725	0.014*	
O3	0.0905 (4)	0.7735 (4)	0.8958 (3)	0.0225 (6)	
C3	0.1271 (6)	0.4323 (5)	0.8265 (3)	0.0140 (7)	
H3B	−0.0362	0.4770	0.8021	0.017*	
H3A	0.1358	0.4019	0.9180	0.017*	
C4	0.5119 (6)	0.8308 (5)	0.7941 (3)	0.0159 (7)	
H4B	0.6647	0.7763	0.7801	0.024*	
H4C	0.5475	0.9323	0.8380	0.024*	
H4A	0.4212	0.8512	0.7116	0.024*	
O2	0.4703 (5)	0.6718 (4)	1.0115 (2)	0.0241 (7)	

### Atomic displacement parameters (Å^2^)

**Table:**

	*U*^11^	*U*^22^	*U*^33^	*U*^12^	*U*^13^	*U*^23^
C1	0.0242 (19)	0.0177 (18)	0.0211 (17)	−0.0055 (15)	0.0130 (14)	−0.0007 (15)
Cl1	0.0110 (4)	0.0182 (5)	0.0161 (4)	−0.0006 (3)	0.0051 (3)	0.0047 (3)
S1	0.0117 (4)	0.0156 (5)	0.0085 (4)	−0.0016 (3)	0.0030 (3)	−0.0020 (3)
O1	0.0205 (13)	0.0198 (14)	0.0186 (12)	−0.0093 (10)	0.0134 (9)	−0.0075 (11)
N1	0.0094 (13)	0.0128 (15)	0.0118 (13)	−0.0016 (11)	0.0051 (10)	−0.0004 (11)
C2	0.0117 (15)	0.0138 (17)	0.0110 (15)	0.0003 (13)	0.0052 (12)	0.0003 (13)
O3	0.0138 (12)	0.0264 (14)	0.0283 (14)	0.0026 (11)	0.0088 (10)	−0.0058 (11)
C3	0.0120 (15)	0.0159 (17)	0.0152 (17)	−0.0047 (15)	0.0076 (12)	−0.0014 (16)
C4	0.0158 (16)	0.0165 (17)	0.0158 (16)	−0.0032 (15)	0.0044 (12)	0.0020 (15)
O2	0.0278 (14)	0.0330 (17)	0.0107 (12)	−0.0060 (12)	−0.0040 (9)	0.0035 (11)

### Geometric parameters (Å, º)

**Table:**

C1—C2	1.515 (5)	N1—H1D	0.8900
C1—H1A	0.9600	N1—H1E	0.8900
C1—H1B	0.9600	N1—H1F	0.8900
C1—H1C	0.9600	C2—C3	1.515 (5)
S1—O3	1.427 (3)	C2—H2A	0.9800
S1—O2	1.430 (3)	C3—H3B	0.9700
S1—O1	1.571 (3)	C3—H3A	0.9700
S1—C4	1.744 (4)	C4—H4B	0.9600
O1—C3	1.468 (4)	C4—H4C	0.9600
N1—C2	1.491 (4)	C4—H4A	0.9600
			
C2—C1—H1A	109.5	N1—C2—C1	110.1 (3)
C2—C1—H1B	109.5	N1—C2—C3	109.5 (3)
H1A—C1—H1B	109.5	C1—C2—C3	110.3 (3)
C2—C1—H1C	109.5	N1—C2—H2A	109.0
H1A—C1—H1C	109.5	C1—C2—H2A	109.0
H1B—C1—H1C	109.5	C3—C2—H2A	109.0
O3—S1—O2	116.97 (15)	O1—C3—C2	105.7 (2)
O3—S1—O1	109.32 (15)	O1—C3—H3B	110.6
O2—S1—O1	108.65 (17)	C2—C3—H3B	110.6
O3—S1—C4	111.10 (17)	O1—C3—H3A	110.6
O2—S1—C4	110.34 (16)	C2—C3—H3A	110.6
O1—S1—C4	98.91 (16)	H3B—C3—H3A	108.7
C3—O1—S1	117.07 (19)	S1—C4—H4B	109.5
C2—N1—H1D	109.5	S1—C4—H4C	109.5
C2—N1—H1E	109.5	H4B—C4—H4C	109.5
H1D—N1—H1E	109.5	S1—C4—H4A	109.5
C2—N1—H1F	109.5	H4B—C4—H4A	109.5
H1D—N1—H1F	109.5	H4C—C4—H4A	109.5
H1E—N1—H1F	109.5		

### Hydrogen-bond geometry (Å, º)

**Table:**

*D*—H···*A*	*D*—H	H···*A*	*D*···*A*	*D*—H···*A*
N1—H1*E*···Cl1	0.89	2.33	3.169 (3)	156
C3—H3*A*···O3^i^	0.97	2.58	3.428 (4)	147
N1—H1*D*···Cl1^ii^	0.89	2.24	3.116 (3)	169
N1—H1*F*···Cl1^iii^	0.89	2.26	3.139 (3)	171
C2—H2*A*···O2^iv^	0.98	2.44	3.186 (4)	133
C4—H4*B*···O3^v^	0.96	2.50	3.250 (4)	135
C4—H4*C*···O2^vi^	0.96	2.51	3.438 (5)	163

Symmetry codes: (i) −*x*, *y*−1/2, −*z*+2; (ii) −*x*+1, *y*−1/2, −*z*+1; (iii) *x*−1, *y*, *z*; (iv) −*x*+1, *y*−1/2, −*z*+2; (v) *x*+1, *y*, *z*; (vi) −*x*+1, *y*+1/2, −*z*+2.
