# Supplementary figures and images for: Crystal structure of (2R)-1-[(methyl­sulfon­yl)­oxy]propan-2-aminium chloride: a chiral mol­ecular salt
Source: Acta Crystallogr E Crystallogr Commun. 2015 Sep 12;71(Pt 10):o733–4. doi: 10.1107/S2056989015015972 (PMC4647372; doi:10.1107/S2056989015015972)

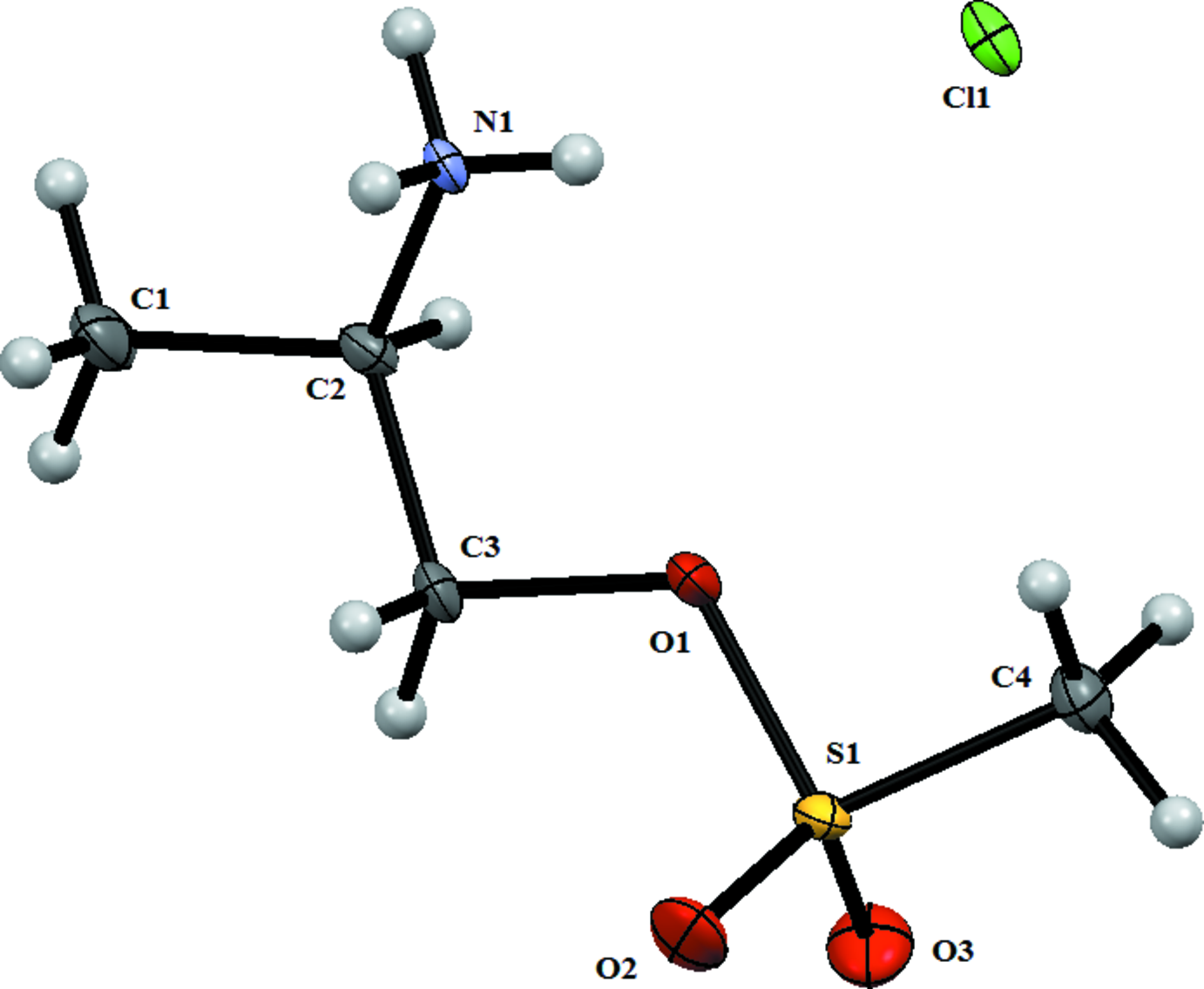

Supplement: Supplementary file 4 [file e-71-0o733-fig1.tif]

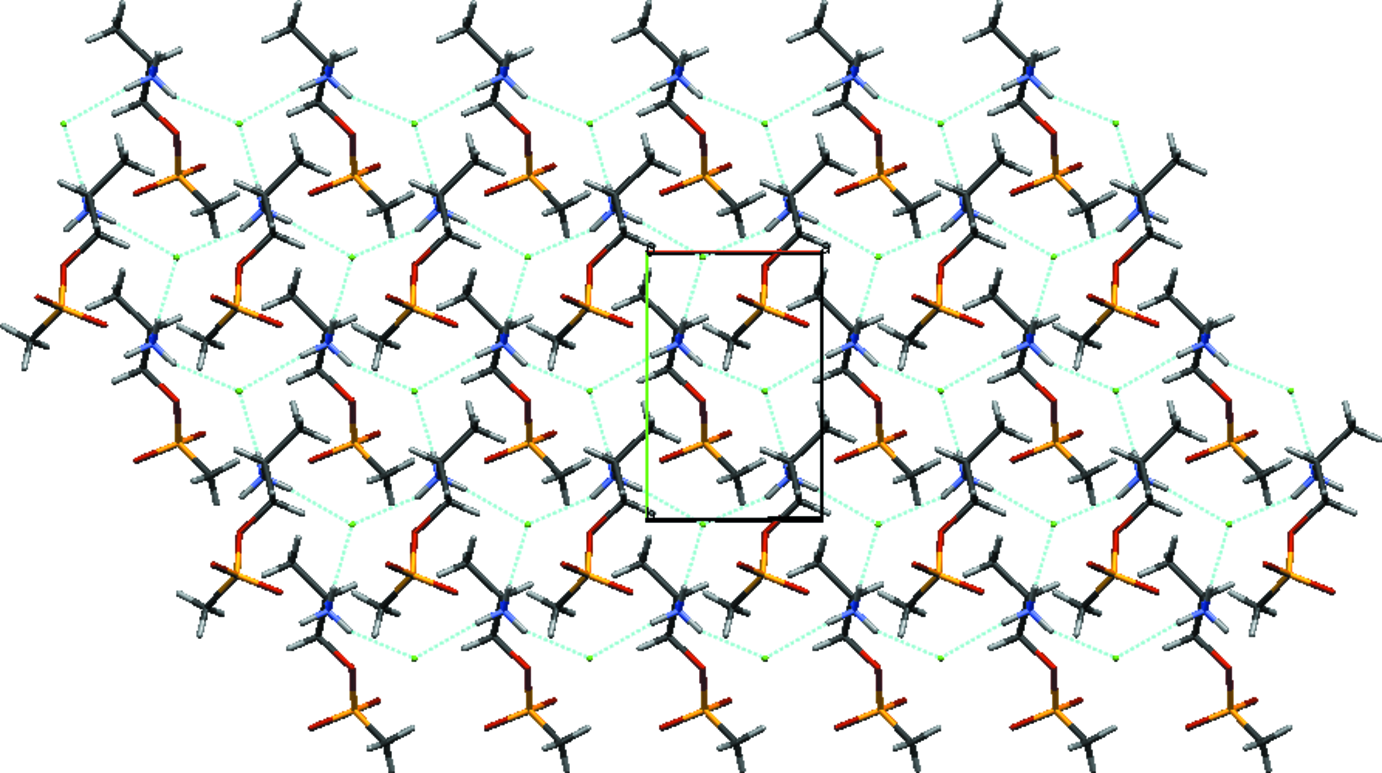

Supplement: Supplementary file 5 [file e-71-0o733-fig2.tif]
